# Associations of health literacy with socioeconomic position, health risk behavior, and health status: a large national population-based survey among Danish adults

**DOI:** 10.1186/s12889-020-08498-8

**Published:** 2020-04-28

**Authors:** Majbritt Tang Svendsen, Carsten Kronborg Bak, Kristine Sørensen, Jürgen Pelikan, Signe Juul Riddersholm, Regitze Kuhr Skals, Rikke Nørmark Mortensen, Helle Terkildsen Maindal, Henrik Bøggild, Gitte Nielsen, Christian Torp-Pedersen

**Affiliations:** 1 Department of Cardiology, North Denmark Regional Hospital, Bispensgade 37, 9800 Hjørring, Denmark; 2 Centre for Clinical Research, North Denmark Regional Hospital / Clinical Institute of Medicine, Aalborg University , Hjørring, Denmark; 3 Department of Research and Development, University College South, Kolding, Denmark; 4 Global Health Literacy Academy, Risskov, Denmark; 5 Austrian Public Health Institute, Vienna, Austria; 6 Department of Anesthesia and Intensive Care, Aalborg University Hospital, Aalborg, Denmark; 7 Epidemiology and Biostatistics, Aalborg University Hospital, Aalborg, Denmark; 8 Department of Public Health, Section for Health Promotion and Health Services, Aarhus University, Aarhus, Denmark; 9 Public Health and Epidemiology Group, Department of Health Science and Technology, Aalborg University, Aalborg, Denmark

**Keywords:** Health literacy, Socioeconomic position, Health behavior, Health status, Health inequality, Social position, Health risk indicators, Population survey, Health literacy questionnaire, HLS-EU-Q16

## Abstract

**Background:**

Health literacy concerns the ability of citizens to meet the complex demands of health in modern society. Data on the distribution of health literacy in general populations and how health literacy impacts health behavior and general health remains scarce. The present study aims to investigate the prevalence of health literacy levels and associations of health literacy with socioeconomic position, health risk behavior, and health status at a population level.

**Methods:**

A nationwide cross-sectional survey linked to administrative registry data was applied to a randomly selected sample of 15,728 Danish individuals aged ≥25 years. By the short form HLS-EU-Q16 health literacy was measured for the domains of healthcare, disease prevention, and health promotion. Adjusted multinomial logistic regression analyses were used to estimate associations of health literacy with demographic and socioeconomic characteristics, health risk behavior (physical activity, smoking, alcohol consumption, body weight), and health status (sickness benefits, self-assessed health).

**Results:**

Overall, 9007 (57.3%) individuals responded to the survey. Nearly 4 in 10 respondents faced difficulties in accessing, understanding, appraising, and applying health information. Notably, 8.18% presented with inadequate health literacy and 30.94% with problematic health literacy. Adjusted for potential confounders, regression analyses showed that males, younger individuals, immigrants, individuals with basic education or income below the national average, and individuals receiving social benefits had substantially higher odds of inadequate health literacy. Among health behavior factors (smoking, high alcohol consumption, and inactivity), only physical behavior [sedentary: OR: 2.31 (95% CI: 1.81; 2.95)] was associated with inadequate health literacy in the adjusted models. The long-term health risk indicator body-weight showed that individuals with obesity [OR: 1.78 (95% CI: 1.39; 2.28)] had significantly higher odds of lower health literacy scores. Poor self-assessed health [OR: 4.03 (95% CI: 3.26; 5.00)] and payments of sickness absence compensation benefits [OR: 1.74 (95% CI: 1.35; 2.23)] were associated with lower health literacy scores.

**Conclusions:**

Despite a relatively highly educated population, the prevalence of inadequate health literacy is high. Inadequate health literacy is strongly associated with a low socioeconomic position, poor health status, inactivity, and overweight, but to a lesser extent with health behavior factors such as smoking and high alcohol consumption.

## Background

During the past decades, an unsolicited socioeconomic divide has intensified in Europe, and the population is increasingly challenged with the growing complexity of the modern healthcare system and the rising expectations of the individuals to participate actively in promoting and maintaining their health [1–3]. The socioeconomic divide is not only about income. It remains a complex phenomenon involving health status which is also driven by education, employment status, and migration background [1]. Health literacy, a multidimensional concept covering the interacting capacities of the individuals and the systems to meet the complex demands of health in modern society, has been recognized as a key factor for reducing health inequality [3]. Despite the enormous implications of inadequate health literacy, knowledge of health literacy in the general population and how health literacy impacts health behavior and health status remain scarce [4].

In recent years, health literacy has gained importance on the European health agenda, and there has been a growing interest in the concept, accompanied by the increased emphasis on the role and responsibilities of the individual in health and healthcare [5–7]. Health literacy research has developed from investigations of general literacy or functional health literacy to expanded conceptual frameworks encompassing citizens´ knowledge, motivation, and competences to access, understand, appraise, and apply health information in everyday life to make decisions regarding healthcare, disease prevention, and health promotion [3, 8]. Comprehensive measurement tools reflecting the multidimensional concept of health literacy have been developed, and a health literacy survey performed by the European Health Literacy Consortium (HLS-EU) in eight member states of the EU in 2011 suggested that nearly half of the European population included in this survey had limited or suboptimal health literacy competences [3, 6, 8–11].

In modern society, individuals with higher health literacy have advantages in health compared with those who have lower health literacy. Several studies have shown that limited health literacy is associated with adverse health-related outcomes, such as increased mortality [12, 13], more hospitalizations [14, 15], less use of preventive care [16], less efficient use of access to health services [17], and the lack of the ability to make informed health-related decisions [18] and follow medical instructions [19]. Limited health literacy has also been demonstrated in European countries to follow a social gradient [9, 11], and the increasing demands on the individual seem inadvertently to raise social disparity in health as it favors those with adequate health literacy [20].

Beyond HLS-EU, the majority of previous research on health literacy is based on smaller samples, focusses on functional or specific dimensions of health literacy, or is centered on a specific population or patient groups [2, 21–25]. There is a growing need to understand how health literacy, as a dynamic outcome of sociodemographic determinants as well as individual and environmental factors, manifests itself in the interaction between individuals and the modern health society at the population level [26]. Comprehensive knowledge of health literacy in the general population is needed for guiding health systems and health organizations in their response to the needs of the citizens.

To the best of our knowledge, the present study constitutes the largest national population-based study on health literacy using the short form (HLS-EU-Q16) of the comprehensive European Health Literacy Survey Questionnaire. Specifically, the study aims to examine the distribution of health literacy levels and investigate the association of health literacy with socioeconomic position, health risk behavior, and health status within a large representative sample of 15, 728 Danish adults.

## Methods

### Study design and sampling

A national, cross-sectional, representative survey with a random stratified sampling design was conducted involving Danish residents aged 25 years and older. A minimum age of 25 years was set in order to obtain a more stable life situation of participants with respect to socioeconomic indicators. Participants were drawn from the Danish Civil Registration System, and the sample was stratified according to age, gender, and geographical location (postal codes), leading to a probability of inclusion that was proportional to population size and density. Postal codes were included to ensure representative sampling from urban and rural residential areas. The sample was individually linked with information obtained from a variety of Danish administrative registries.

### Study population

Between December 2016 and February 2017, 15,728 citizens were contacted; of these, 9007 were willing to participate, leading to a response rate of 57.3%. Participants were recruited using an electronic email system administered by public authorities including a link to a self-administered web-based questionnaire. The wording of the material was provided only in Danish and was not specifically targeted to low-literate individuals. A random part of the study population (*N* = 1082) were contacted by telephone to increase representation.

### Measures

Overall, the survey instrument comprised 28 items divided into the following categories: (1) health literacy, (2) health behavior, (3) health risk indicators, and (4) perceived self-assessed health. Data on demographic characteristics, socioeconomic indicators, and health status were obtained from nationwide administrative registries.

#### Health literacy

The HLS-EU-Q16 questionnaire developed by the HLS-EU Consortium for measuring health literacy in the general population was applied. Extensive information on the development and pre-testing is described elsewhere [4, 8]. To ensure cross-cultural validity, translation and adaption of the Health Literacy Survey Questionnaire followed a standardized procedure completed by K. Sørensen, H. Maindal, and colleagues (Unpublished material. Please contact the third author, K. Sørensen, for further information). The short form of the HLS-EU-Q used in this present study consisted of 16 items measuring health literacy within the three domains of healthcare, disease prevention, and health promotion. Within these domains, questions focus on perceived difficulties or ease of accessing, understanding, appraising, and applying health information [4, 27]. Each of the items had response categories on a 4-point Likert scale ranging from “very easy” to “very difficult.” Health literacy scores ranging from 0 to 16 were calculated by dichotomizing answer categories. “Very easy” and “easy” were given a score of 1, whereas “very difficult” and “difficult” were given a score of 0. The total health literacy score was classified according to three levels, namely inadequate, (0–8), problematic (9–12), and adequate (13–16). A “don’t know” answer option was provided in the telephone interviews and was used only when stated by the respondent spontaneously. The “don’t know” category was coded as a missing value. Health literacy scores were computed only for respondents who had answered a minimum of 14 of all health literacy items [4], corresponding to a total of 8455 (93.9%) respondents.

#### Demographic and socioeconomic characteristics

In Denmark, all citizens are identified with a unique civil registration number that enables individual linkage of information across Danish administrative registries. We retrieved information on age (year of birth), gender (male, female), origin (Danish, immigrant, descendant of immigrant), marital status (married/partnership, divorced, unmarried), and residence from the Danish Civil Personal Registration Registry [28]. Information on education, described using the International Standard Classification of Education (ISCED) nomenclature, was obtained from the Danish Education Registers [29]. Educational level was grouped into categories based on highest completed education level one year before the survey completion. Basic school, primary educations, lower secondary (ISCED 0–2), upper secondary, and vocational educations (ISCED 3–4) reflect the first and second education levels. Medium length educations including short and medium length tertiary, and bachelor’s educations (ISCED 5–6), and higher length education containing master’s level and PhD-level educations (ISCED 7–8) reflect the third and fourth levels of education. Annual income was obtained from the Danish Income Register [30] and was calculated as the mean of the respondents’ individual average income in the last three years before the survey completion. Based on the average income of 312,000 DKK [31] in the general Danish population (7.45 DKK equals 1 €), income was divided in two groups: below average (< 312,000 DKK) or above average (≥312,000 DKK). Finally, information on social benefits was obtained from a registry administered by the Danish Labour Market Authority (the DREAM database) [32]. Maternity leave compensation and sickness compensation were not considered social benefits.

#### Health behavior, health risk indicators, and health status

Different measures of health behavior including smoking habits, alcohol consumption, and physical activity were included from the survey. Smoking was classified as “daily smoker”, “infrequent smoker”, “former smoker”, or “never smoker”. Alcohol consumption was measured according to official national recommendations stated by the Danish Health Authorities. Weekly alcohol consumption above 14 drinks for men and seven drinks for women is considered high-risk behavior. Physical activity was classified according to daily physical activity level as “sedentary behavior”, “light activities”, “moderate training”, or “hard training” within the last year before the survey completion.

Self-reported height and weight were obtained to allow calculation of body mass index (BMI). BMI was regarded as a health risk indicator and classified as underweight (BMI ≤18.5 kg/m^2^), normal (BMI 18.5–24.9 kg/m^2^), overweight (BMI 25–30 kg/m^2^), or obese (BMI ≥30 kg/m^2^). Self-assessed general health stated as “How would you judge your current state of health?” included four categories ranging from very good to very poor. Information on sickness absence compensation benefits one year from the survey completion was obtained from the Dream database.

### Statistical analysis

The categorical variables are presented using percentages and the continuous variables using medians with the 25th (Q1) and 75th (Q3) percentiles. Chi-square and Kruskal-Wallis tests were performed to test differences between respondents and non-respondents as well as differences between health literacy groups. Internal consistency and reliability of health literacy levels were assessed calculating Cronbach’s alpha coefficients based on Pearson correlations. The level of perceived difficulty was calculated in terms of health literacy competences and domains (sum score/number of items) using means with standard deviations and medians with the 25th (Q1) and 75th (Q3) percentiles. We estimated individual odds ratios (OR) between levels of health literacy (outcome variable) and demographic and socioeconomic measures, health risk behavior, and health status (exposure variables) using both univariable and multivariable multinomial logistic regression analyses compared to odds of adequate health literacy. Demographic and socioeconomic factors, including gender, age, migration background, civil status, education, and income were included as potential confounders in the regression analyses. We tested for interaction using the likelihood ratio test. The reference groups in the models were consistently chosen as the more prevalent ones. Sensitivity analyses were performed to test the consistency of the general health literacy score according to the method of distribution of survey material, including either web-based or telephone interview-based. A two-sided *P*-value < 0.05 was *considered* statistically significant. Statistical analyses were performed by the statistical software packages SAS version 9.4 (SAS Institute Inc., Cary, NC, USA), and R statistical software package, version 3.3.2 (R Development Core Team) [33].

### Ethics

According to Danish legislation (Law on ethical conduct in health science, Lovtidende:§14, section 2) application for ethical approval is not required for questionnaire-based and register-based studies [34]. The written provision of information about the survey communicated to participants, including information on data retrieval along with the voluntary completion by participants, constituted an implied consent. The data collection was approved by the Danish Data Protection Agency (j.no: 2008-58-0028) and was conducted in accordance with the Helsinki Declaration.

## Results

A total of 9007 residents (57.3%) participated in the survey. The median age of respondents was 53.2 years [Q1: 42.3, Q3: 63.7], slightly more women (54.5%) than men participated, and 7.2% were immigrants or descendants of immigrants. The majority of respondents were married (64.0%), approximately one-third (28.9%) had an annual income below the national average income, and more than one-fifth (21.9%) of participants received social welfare payments within the last year from the survey completion. Characteristics of respondents and non-respondents according to sociodemographic indicators are presented in Table 1. The sociodemographic characteristics were distributed differently between respondents and non-respondents (*P < 0.001*), and especially the youngest age group was underrepresented.

**Table 1:** Sociodemographic characteristics of respondents versus non-respondents

	Respondents (*n* = 9007)	Non-respondents (*n* = 6721)	Total (*n* = 15,728)	*P*-value
**Sex**				
Female	4913 (54.5)	2963 (44.1)	7876 (50.1)	
Male	4094 (45.5)	3758 (55.9)	7852 (49.9)	< 1e-04
**Age, Median [Q1,Q3]**	53.2 [42.3, 63.7]	44.2 [34.9, 55.2]	49.6 [38.6, 60.7]	< 1e-04
**Age group**				
25–44	2755 (30.6)	3504 (52.1)	6259 (39.8)	
35–54	2175 (24.1)	1507 (22.4)	3682 (23.4)	
55–64	2074 (23.0)	1026 (15.3)	3100 (19.7)	
> 65	2003 (22.2)	684 (10.2)	2687 (17.1)	< 1e-04
**Origin**				
Danish	8357 (92.8)	5498 (81.8)	13,855 (88.1)	
Immigrant	611 (6.8)	1141 (17.0)	1752 (11.1)	
Descendant of immigrant	39 (0.4)	82 (1.2)	121 (0.8)	< 1e-04
**Civil status**				
Married/Partnership	5755 (64.0)	3394 (50.9)	9149 (58.5)	
Divorced	1146 (12.8)	813 (12.2)	1959 (12.5)	
Unmarried	2086 (23.2)	2455 (36.9)	4541 (29.0)	< 1e-04
Missing	20	59	79	
**Education**				
Basic School	1458 (16.6)	1354 (22.2)	2812 (18.9)	
High school/Vocational	3791 (43.3)	2772 (45.4)	6563 (44.1)	
Medium	2474 (28.2)	1345 (22.0)	3819 (25.7)	
High	1035 (11.8)	640 (10.5)	1675 (11.3)	< 1e-04
Missing	249	610	859	
**Income**				
Below average	2606 (28.9)	2588 (38.5)	5194 (33.0)	
Above average	6401 (71.1)	4133 (61.5)	10,534 (67.0)	< 1e-04
**Welfare payments**				
Non-social benefit	4858 (54.4)	3952 (60.1)	8810 (56.8)	
Retirement benefit	2115 (23.7)	714 (10.9)	2829 (18.3)	
Social benefit	1952 (21.9)	1908 (29.0)	3860 (24.9)	< 1e-04
Missing	82	147	229	

*P*resents demographic and socioeconomic characteristics of respondents versus non-respondents (*N* = 15,728) of Danish residents aged 25 years or older in 2016 and 2017. Data are presented as medians with 25th (Q1) and 75 th (Q3) percentiles (age) or number of residents and percentage (all others)

### Distribution of health literacy within the population

The median health literacy score of respondents was 13.0 [*Q1: 11.0, Q3: 15.0*] on the 16-item scale. The health literacy scale was further classified into three levels described previously. Overall, 8.2% (*N = 692*) of the study population had inadequate health literacy, 30.9% (*N = 2616)* had problematic health literacy, and 60.9% (*N = 5147*) showed adequate health literacy. For individuals categorized within the inadequate health literacy category, the median health literacy score was 7.0 [*Q1: 6.0, Q3: 8.0]*. Individuals within the problematic or adequate health literacy category presented with a median score of 11.0 [*Q1: 10.0, Q3: 12.0]* and 14.0 [*Q1: 14.0, Q3: 16.0]*, respectively. The Cronbach alpha coefficient indicated a high internal consistency of the assembled HLS-EU-Q16 questionnaire (α = 0.90) and good or acceptable internal consistency within each of the three health domains (healthcare: α = 0.82, disease prevention: α = 0.74, health promotion: α = 0.75). For the competences of accessing, understanding, appraising, and applying information, the median score per item was highest for accessing health information (Median: 3.3 [*Q1: 3.0, Q3: 3.5*]) and lowest for appraising health information (Median: 2.7 [*Q1: 2.3, Q3: 3.0*]). When comparing all competences over the health domains of healthcare, disease prevention, and health promotion, the median score per item was highest within the domain of healthcare (Median: 3.2 [*Q1: 2.9, Q3: 3.4*]) and lowest in the domain of disease prevention (Median: 2.8 [*Q1: 2.6, Q3: 3.2*]) (Table 2). The items that were rated least and that the respondents perceived most difficult were: ‘*judge if the information on health risks in the media is reliable’* (*N = 4668*, proportion experiencing difficulty: 62.3%) and ‘*judge when you may need to get a second opinion from another doctor*’ (*N = 4173*, proportion experiencing difficulty: 53.3%). In contrast, respondents experienced the least difficulty in relation to: *‘understand your doctor’s or pharmacist’s instruction on how to take a prescribed medicine’* (*N = 169*, proportion experiencing difficulty: 2.0%) and *‘understand health warnings about behavior such as smoking, low physical activity and drinking too much’* (*N = 171*, proportion experiencing difficulty: 2.0%).

**Table 2:** Health literacy by health domains and health competences (*N* = 8455)

	Items	Missings	Mean (SD)	Mean per item (SD)	Median (Q1, Q3)	Median per item (Q1, Q3)
**Health domains**						
Healthcare	7	260	22,21 (3,10)	3,17 (0,44)	22.00 (20.00, 24.00)	3.15 (2.86, 3.43)
Disease prevention	5	181	14,34 (2,47)	2,87 (0,49)	14.00 (13.00, 16.00)	2.80 (2.60, 3.20)
Health promotion	4	88	12,13 (1,97)	3,03 (0,49)	12.00 (11.00, 13.00)	3.00 (2.75, 3.25)
**Health competences**						
Accessing	4	85	12,67 (1,84)	3,17 (0,46)	13.00 (12.00, 14.00)	3.25 (3.00, 3.5.0)
Understanding	6	267	15,29 (2,32)	3,06 (0,46)	15.00 (14.00, 17.00)	3.00 (2.80, 3.40)
Appraising	3	135	8,41 (1,59)	2,80 (0,53)	8.00 (7.00, 9.00)	2.67 (2.33, 3,00)
Applying	3	36	9,21 (1,32)	3,07 (0,44)	9.00 (8.00, 10.00)	3.00 (2.67, 3.33)

Health literacy by health domains and health competences of Danish residents aged 25 years or older in 2016 and 2017. Data are presented as medians with 25th (Q1) and 75th (Q3) percentiles and means with standard deviations (SD)

Overall, health literacy varied between subgroups according to demographic and socioeconomic characteristics within the population (Table 3). Men, younger aged individuals (25–44 years old), non-ethnic Danes, unmarried individuals, people with a low education level, income below the national average, and individuals receiving public benefits reported statistically significantly lower levels of health literacy (*P < 0.001*). The sensitivity analysis concerning the method of distribution of survey material showed that the general health literacy score was slightly lower among interview-based respondents (Median: 12.0 [*Q1: 11.0, Q3: 14.0*]) compared to web-based respondents (Median: 13.0 [*Q1: 11.0, Q3: 15.0*]). Socioeconomic characteristics and general health literacy score of interview-based respondents compared to web-based respondents are available in data supplement (Table S1).

**Table 3:** Sociodemographic characteristics by general health literacy

	Inadequate (*n* = 692)	Problematic (*n* = 2616)	Adequate (*n* = 5147)	Total (*n* = 8455)	*P*-value
**Sex**					
Female	280 (40.5)	1319 (50.4)	3043 (59.1)	4642 (54.9)	
Male	412 (59.5)	1297 (49.6)	2104 (40.9)	3813 (45.1)	< 1e-04
**Age, Median [Q1,Q3]**	51.3 [39.6, 62.1]	52.6 [40.9, 63.3]	53.9 [43.4, 64.2]	53.3 [42.4, 63.8]	< 1e-04
**Age group**					
25–44	251 (36.3)	862 (33.0)	1438 (27.9)	2551 (30.2)	
35–54	156 (22.5)	618 (23.6)	1282 (24.9)	2056 (24.3)	
55–64	157 (22.7)	578 (22.1)	1227 (23.8)	1962 (23.2)	
> 65	128 (18.5)	558 (21.3)	1200 (23.3)	1886 (22.3)	< 1e-04
**Origin**					
Danish	618 (89.3)	2420 (92.5)	4818 (93.6)	7856 (92.9)	
Immigrant	69 (10.0)	180 (6.9)	314 (6.1)	563 (6.7)	
Descendant of immigrant	5 (0.7)	16 (0.6)	15 (0.3)	36 (0.4)	0.0001
**Civil status**					
Married/Partnership	402 (58.3)	1608 (61.7)	3400 (66.1)	5410 (64.1)	
Divorced	84 (12.2)	327 (12.5)	675 (13.1)	1086 (12.9)	
Unmarried	203 (29.5)	672 (25.8)	1066 (20.7)	1941 (23.0)	< 1e-04
Missing	3	9	6	18	
**Education**					
Basic School	140 (21.3)	445 (17.4)	776 (15.5)	1361 (16.5)	
High school/Vocational	325 (49.4)	1130 (44.3)	2097 (41.8)	3552 (43.2)	
Medium	133 (20.2)	670 (26.3)	1543 (30.8)	2346 (28.5)	
High	60 (9.1)	307 (12.0)	600 (12.0)	967 (11.8)	< 1e-04
Missing	34	64	131	229	
**Income**					
Below average	272 (39.3)	817 (31.2)	1332 (25.9)	2421 (28.6)	
Above average	420 (60.7)	1799 (68.8)	3815 (74.1)	6034 (71.4)	< 1e-04
**Welfare payments**					
Non-social benefit	318 (46.6)	1376 (53.0)	2849 (55.8)	4543 (54.2)	
Retirement benefit	137 (20.1)	595 (22.9)	1267 (24.8)	1999 (23.9)	
Social benefit	228 (33.4)	623 (24.0)	988 (19.4)	1839 (21.9)	< 1e-04
Missing	9	22	43	74	

Presents general health literacy scores by health literacy level (*N* = 8455) according to demographic and socioeconomic indicators of Danish residents aged 25 years or older in 2016 and 2017. Data are presented as medians with 25th (Q1) and 75th (Q3) percentiles (age) or number of residents and percentage (all others)

### Associations of health literacy with demographic and socioeconomic characteristics

**Fig. 1:**
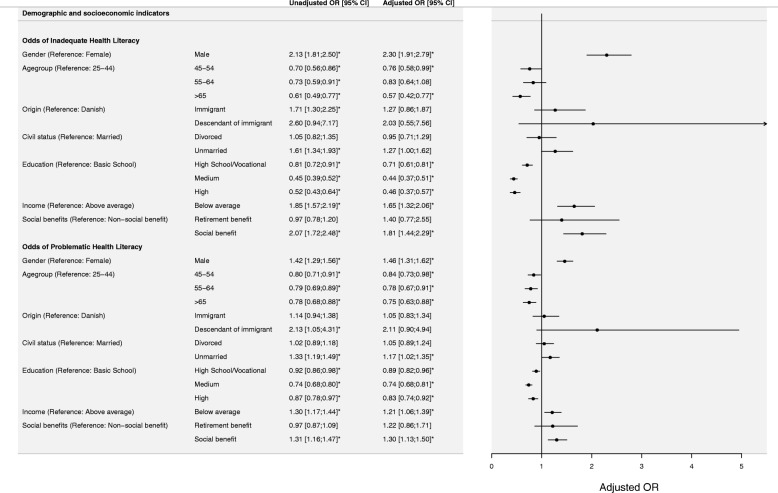
Associations of demographic and socioeconomic characteristics with health literacy. Forrest plot presenting multivariable multinomial logistic regression model describing odds ratios (OR), with corresponding 95% confidence intervals (CI), of inadequate and problematic health literacy compared to adequate health literacy. Unadjusted and model adjusted for all covariates. Statistically significant *P*-values (*P < 0.05*) are flagged with star symbols (*)

Figure 1 shows odds ratios and confidence intervals for both univariable and multivariable logistic multinomial regression analysis estimating the odds of having inadequate health literacy compared to adequate health literacy, and further problematic health literacy compared to adequate health literacy. Males had significantly higher odds of inadequate [Adjusted OR: 2.30 (95% CI: 1.91; 2.79)] and problematic [Adjusted OR: 1.46 (95% CI: 1.31; 1.62)] health literacy compared to women. The odds of experiencing both inadequate and problematic health literacy diminished with higher age. The socioeconomic indicators, adjusted for covariates, showed that migration background, education, income, and transfer of public benefits were statistically significantly associated with health literacy. Individuals with high school or vocational, medium or high education had significantly lower odds of inadequate and problematic health literacy compared to an individual with only primary or basic education (ISCED level 0–4) as highest completed education level. Individuals with an annual income below the national average had higher odds of inadequate and limited health literacy. The adjusted multinomial logistic regression model showed that individuals receiving social benefits tend to have lower general health literacy scores compared to individuals who are self-supporting or receiving retirement benefits.

### Associations of health literacy with health behavior and health risk indicators

**Fig. 2:**
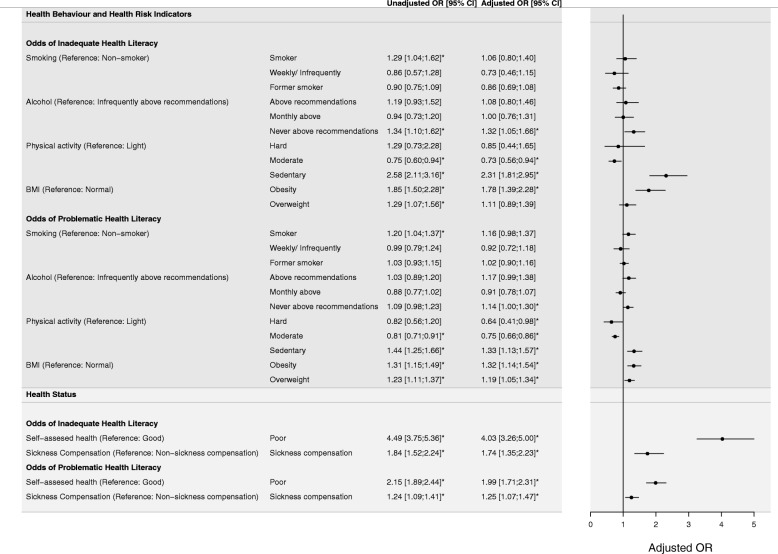
Associations of health risk behavior and health status with health literacy. Forrest plot presenting multivariable multinomial logistic regression model describing odds ratios (OR), with corresponding 95% confidence intervals (CI), of inadequate and problematic health literacy compared to adequate health literacy. Unadjusted and model adjusted for all covariates. Statistically significant P-values (*P < 0.05*) are flagged with star symbols (*)

The association between health literacy and health behavior (Fig. 2) showed no associations of inadequate health literacy with smoking and alcohol consumption above national recommendations in the adjusted models. Alcohol consumption “never above recommendations” was associated with higher odds of inadequate health literacy*.* Significant associations were found between health literacy and physical activity. Individuals reporting sedentary behavior had higher odds of lower general health literacy scores compared to individuals reporting light activities as a physical behavior pattern. Contrarily, individuals reporting moderate exercise behavior had lower odds of both inadequate and problematic health behavior. Significant associations in both univariable and multivariable models with the long-term health risk indicator BMI were found. Obesity (BMI > 30) was associated with lower general health literacy scores [Inadequate health literacy: Adjusted OR: 1.78 (95% CI: 1.39; 2.28), problematic health literacy: Adjusted OR: 1.32 (95% CI: 1.14; 1.54)]. Significant associations with health literacy and overweight (BMI > 25) were also found, demonstrating that individuals with higher health literacy scores tend to have a normal BMI.

### Health literacy and health status

A large proportion of respondents reported their health as good (64.7%) or very good (19.0%), compared to individuals reporting their health as poor (14.9%) or very poor (1.4%). A strong association between both inadequate [Adjusted OR: 4.03 (95%CI: 3.26; 5.00)] and problematic [Adjusted OR: 1.99 (95%CI: 1.71; 2.31)] health literacy with poor and very poor self-assessed health was found (Fig. 2), demonstrating that individuals reporting poor or very poor self-assessed health are more likely to have lower health literacy*.* Payment of sickness absence compensation benefits was used as a proxy for health status. In both univariable and multivariable regression models, significant associations between lower health literacy scores and payments of sickness absence compensation benefits were found (Fig. 2*)*.

## Discussion

Nearly four in 10 of the Danish population reports having difficulties managing and meeting the complex demands of health. Gender, age, ethnicity, education, income, and transfer of public payments were all associated with health literacy levels. Health literacy was strongly associated with physical activity, body weight, self-assessed health, and payments of sickness absence compensation, but not with smoking and to a lesser extent with alcohol consumption.

The present study is the first nationwide representative population study of health literacy in Denmark using the HLS-EU-Q16. Previous studies on health literacy were based on regional data and focused on specific dimensions of health literacy [23] or specific target groups [24, 25]. Health literacy was measured on an international validated instrument, and to our knowledge, this is the largest sample applied in a single study using the HLS-EU-Q16. The study provided information on an individual’s self-perceived competences necessary for them to make empowered and informed decisions regarding health, reflected by the competences of accessing, understanding, appraising, and applying information in the domains of healthcare, disease prevention, and health promotion. The findings of this study indicate that the Danish population perceives the least difficulties within the domain of healthcare and that nearly every second respondent faced problematic or inadequate health literacy. This is consistent with findings from eight other European countries where 29% and 62% of the population (average: 47.6%) were categorized as having limited (inadequate or problematic) health literacy [9, 11]. The health literacy survey of Dutch adults also showed that the mean score per item (over all domains) was lowest for appraising information, which is also consistent with findings in the Danish sample [35]. The general HL-score varied considerably between participating countries in the HLS-EU project.

Though the average health literacy score of the Danish population was within the range of other European countries, it seems that the general Danish population perceives lesser difficulties compared to the majority of other participating countries in the HLS-EU project, but this may reflect that the 16-item scale may have an overrepresentation of easier items than the 47-item scale. Given that the present study was conducted in a country with a universal healthcare system and multiple policies promoting health efforts among the general population, it is disturbing that a substantial part of the population experiences difficulties in making empowered and informed decisions regarding health. Besides the present study, another study using the HLS-EU-Q16 instrument on a national sample has been identified [36]. High internal consistency was found in both the Danish and the Israeli population, indicating that the 16-item scale is reliable and can be used instead of the larger 47-item scale. Inadequate health literacy competences were less pronounced in the Israeli population compared to both the Danish sample and the other eight European countries included in the HLS-EU project [9, 11]. Our results may reflect the increasing complexity of being a health literate individual navigating in modern health society.

The analyses indicate that lower health literacy is associated with lower socioeconomic position, which is in accordance with existing literature [9, 11, 35, 37]. The socioeconomic gradient in health literacy found in the present study is similar to the results of the HLS-EU survey in which health literacy is dependent on socioeconomic indicators such as social status, education, and financial resources [9, 11]. The reason for this association is not well understood and still needs to be explored prospectively, as it may be critical in understanding the relationship to the socioeconomic divide and health inequalities. Interestingly, the present study indicated lower health literacy among the younger population groups, which is in contrast to results from the European countries of the HLS-EU study [9, 11]. Mixed results regarding the association of age and health literacy have previously been discussed by van der Heide et al., who found that age is associated with lower health literacy within specific health literacy dimensions [35]. A previous survey on health literacy performed in Denmark using another health literacy questionnaire (HLQ™) also found that individuals aged 25–45 years perceive more difficulties with health literacy compared to older individuals [23]. They suggested that older individuals may have strengthened capabilities as a result of a more established relationship with their general practitioner and longer experience in navigating in the healthcare sector. The fact that the elderly population in Denmark is relatively highly educated could also contribute to the contradicting results of the relation of age to health literacy.

Another finding of this study showed that men perceive significantly more difficulties with health literacy compared to women with more than twice the odds of problematic health literacy. No consistent pattern between gender and health literacy has been reported in the literature. However, the finding of modest differences between men and women depending on specific health literacy dimensions has previously been reported [23, 35]. Contrarily, the present study showed a strong association between health literacy and gender independent of specific health literacy dimensions.

Similar to the HLS-EU survey, the present study showed a positive association between health literacy and health-related behavior in the form of smoking, alcohol consumption, physical activity level, and body weight [9, 11]. Yet, when controlling for socioeconomic factors; only physical activity and body weight were associated with health literacy. These results imply that although adequate health literacy competences help gain access to appropriate sources of health information critical for the adoption of health behavior, they are not the only factors influencing health behavior. In accordance with existing literature, a strong and positive association of health literacy with self-assessed health was found [9, 36]. The present study supports those results from the other eight European countries of the HLS-EU study that found an association between health literacy and self-assessed health beyond sociodemographic and behavioral measures. Longitudinal data are needed to understand the exact relationship and pathways in which these two variables interact with each other. To our knowledge, no other studies have used payments of sickness absence compensation as a proxy for health status in relation to health literacy. A clear association was found, indicating that health literacy is strongly associated with health status. The significant relation of health literacy with health risk indicators and health status implies consistent attention to the risk of non-communicable diseases (NCDs) within the health system and society at large. The study reveals a triple burden for people with limited health literacy as there is a strong association between being poor, having poor health status, and poor levels of health literacy. These results are in line with previous European studies [11].

The strong socioeconomic divide remains a barrier for people to achieve and maintain good health, also in a welfare state such as Denmark. The divide calls for action in terms of targeted interventions that serve the specific need of people with insufficient and limited health literacy. While almost 40% of the population is challenged in terms of accessing, understanding, appraising, and applying information to manage their health in everyday life, universal health literacy precautions are recommended to facilitate a better match between people’s needs and the services and information offered through the health system [38].

### Strengths and limitations

To our knowledge, the present study is the largest health literacy study of individual respondents using the HLS-EU-Q16 within a single country. A clear strength of this study is, therefore, the large sample size which allowed us to perform wide-ranging and robust investigations of health literacy across demographic, socioeconomic, and health-related indicators. Secondly, the use of nationwide administrative registries is an important strength. Thereby, we were able to adjust our analyses for a wide range of socioeconomic factors and other potential confounders. Another strength of using administrative registries is that we did not rely predominantly on self-reported information concerning socioeconomic indicators. Self-reported data could otherwise result in imprecision and biased results. Thirdly, a major advantage is the use of a short validated health literacy measurement tool, which is relatively easy to administer and allows for comparison with other population groups. However, a drawback of self-reporting questionnaires is that it requires a certain level of literacy and motivation to participate. We suspect that the most vulnerable population groups may not have participated in the study, but the study had the possibility to describe non-responders concerning their socioeconomic status.

Given that non-responders were differently distributed across sociodemographic determinants compared to responders, sensitivity analyses have been performed to evaluate the potential influence of selection bias. We examined the effect of having a web-based reporting tool. The general health literacy score was slightly lower among telephone interview-based respondents compared to web-based respondents, which at least partly can be explained by differences in distributions of the two samples of some factors related to health literacy like gender, age, education, and social benefits. Therefore, overestimation of the general population’s health literacy is possible when using a web-based collection of information. Further studies collecting information on health literacy in different ways or settings are recommended. A limitation is the cross-sectional design that precludes any causal conclusions. Longitudinal studies may provide a better basis to understand these aspects, especially regarding how health literacy may act as a mediator between social determinants and health. Further, the odds estimated in the present study could possibly exaggerate the true effect [39].

### Implications

The evidence from the present study is important for shaping future health and healthcare in Denmark and other welfare societies. To bridge the gap of inequality, solutions need to be developed tackling the triple burden related to health that some population groups encounter. A systematic, organizational change using personalized approaches is required to overcome the barriers. Collaborative efforts are needed within all sectors regarding policy, research, practice, and education.

## Conclusions

Despite a relatively educated population in Denmark, the prevalence of inadequate and problematic health literacy is high in our study. Notably, males, younger individuals, immigrants, individuals with basic education or income below the national average, and individuals receiving social benefits had a substantially higher risk of inadequate health literacy. An independent association between low socioeconomic position and low health literacy was demonstrated. Likewise low health literacy was associated with poor self-reported health, receiving sickness benefits, and with inactivity, but not with smoking and alcohol consumption. Finally, low health literacy was associated with overweight. A significant proportion of the general population faces serious problems in managing health demands. These findings emphasize that universal health literacy precautions are needed to facilitate a better match between people’s needs and the services and information offered through the Danish health system.

## Supplementary information

**Additional file 1: Table S1.** Demographic and socioeconomic characteristics of Danish residents aged 25 years or older in 2016 and 2017 by interview or web-based distribution. Data are presented as medians with 25th (Q1) and 75th (Q3) percentiles (age) or number of residents and percentage (all others).

## Data Availability

The datasets used and/or analysed during the current study are available from the corresponding author on reasonable request.

## Abbreviations

HLS-EU: Health Literacy Survey
HLQ™: Health Literacy Questionnaire
BMI: Body mass index
Q1: 25th percentile
Q3: 75th percentile
OR: Odds ratio
CI: Confidence intervals
SD: Standard deviation
NCDs: Non-communicable diseases
DKK: Danish kroner
€: Euro
ISCED: International Standard Classification of Education
