# Supplementary material for: Associations of health literacy with socioeconomic position, health risk behavior, and health status: a large national population-based survey among Danish adults
Source: BMC Public Health. 2020 Apr 28;20:565. doi: 10.1186/s12889-020-08498-8 (PMC7187482; doi:10.1186/s12889-020-08498-8)
Supplement: Supplementary file 1 — Additional file 1: Table S1. Demographic and socioeconomic characteristics of Danish residents aged 25 years or older in 2016 and 2017 by interview or web-based distribution. Data are presented as medians with 25th (Q1) and 75th (Q3) percentiles (age) or number of residents and percentage (all others). [file 12889_2020_8498_MOESM1_ESM.docx]

| **Table S1. Sociodemographic characteristics and general health literacy scores (*N*=8,455) by interview or web-based survey distribution** | | | | |
| --- | --- | --- | --- | --- |
|  | **Interview-based (n=805)** | **Web-based (n=7,650)** | **Total (n=8,455)** | **p-value** |
| **Health literacy score, median [Q1; Q3]** | 12.0 [11.0, 14.0] | 13.0 [11.0, 15.0] | 13.0 [11.0, 15.0] | < 1e-04 |
| **Health literacy level** |  |  |  |  |
| Inadequate | 55 (6.8) | 637 (8.3) | 692 (8.2) |  |
| Problematic | 358 (44.5) | 2258 (29.5) | 2616 (30.9) |  |
| Adequate | 392 (48.7) | 4755 (62.2) | 5147 (60.9) | < 1e-04 |
| **Sex** |  |  |  |  |
| Female | 414 (51.4) | 4228 (55.3) | 4642 (54.9) |  |
| Male | 391 (48.6) | 3422 (44.7) | 3813 (45.1) | 0.0408389 |
| **Age**, **median [Q1; Q3]** | 49.9 [38.7, 61.2] | 53.6 [42.8, 63.9] | 53.3 [42.4, 63.8] | < 1e-04 |
| **Age group** |  |  |  |  |
| 25-44 | 322 (40.0) | 2229 (29.1) | 2551 (30.2) |  |
| 45-54 | 172 (21.4) | 1884 (24.6) | 2056 (24.3) |  |
| 55-64 | 161 (20.0) | 1801 (23.5) | 1962 (23.2) |  |
| >65 | 150 (18.6) | 1736 (22.7) | 1886 (22.3) | < 1e-04 |
| **Origin** |  |  |  |  |
| Danish | 749 (93.0) | 7107 (92.9) | 7856 (92.9) |  |
| Immigrant | 50 (6.2) | 513 (6.7) | 563 (6.7) |  |
| Descendant of immigrant | 6 (0.7) | 30 (0.4) | 36 (0.4) | 0.3007296 |
| **Civil status** |  |  |  |  |
| Married | 486 (60.5) | 4924 (64.5) | 5410 (64.1) |  |
| Divorced | 97 (12.1) | 989 (13.0) | 1086 (12.9) |  |
| Unmarried | 220 (27.4) | 1721 (22.5) | 1941 (23.0) | 0.0079670 |
| missing | 3 | 16 | 18 |  |
| **Education** |  |  |  |  |
| Basic School | 155 (19.3) | 1206 (15.8) | 1361 (16.1) |  |
| High school/Vocational | 348 (43.2) | 3204 (41.9) | 3552 (42.0) |  |
| Medium | 203 (25.2) | 2143 (28.0) | 2346 (27.7) |  |
| High | 80 (9.9) | 887 (11.6) | 967 (11.4) |  |
| missing | 19 | 210 | 229 | 0.0400232 |
| **Income** |  |  |  |  |
| Below average | 277 (34.4) | 2144 (28.0) | 2421 (28.6) |  |
| Above average | 528 (65.6) | 5506 (72.0) | 6034 (71.4) | 0.0001631 |
| **Welfare payments** |  |  |  |  |
| No social benefit | 442 (55.4) | 4101 (54.1) | 4543 (54.2) |  |
| Retirement benefit | 161 (20.2) | 1838 (24.2) | 1999 (23.9) |  |
| Social benefit | 195 (24.4) | 1644 (21.7) | 1839 (21.9) | 0.0210257 |
| missing | 7 | 67 | 74 |  |

**Table S1.** Demographic and socioeconomic characteristics of Danish residents aged 25 years or older in 2016 and 2017 by interview or web-based distribution. Data are presented as medians with 25th (Q1) and 75th (Q3) percentiles (age) or number of residents and percentage (all others).
